# Legislation Raising the Legal Drinking Age in Massachusetts

**Published:** 1995

**Authors:** Patricia F. Waller

**Affiliations:** Patricia F. Waller, Ph.D.; is the director of the Transportation Research Institute, University of Michigan, Ann Arbor, Michigan

**Keywords:** Massachusetts, legislation, minimum drinking age laws, evaluation

**Figure:**
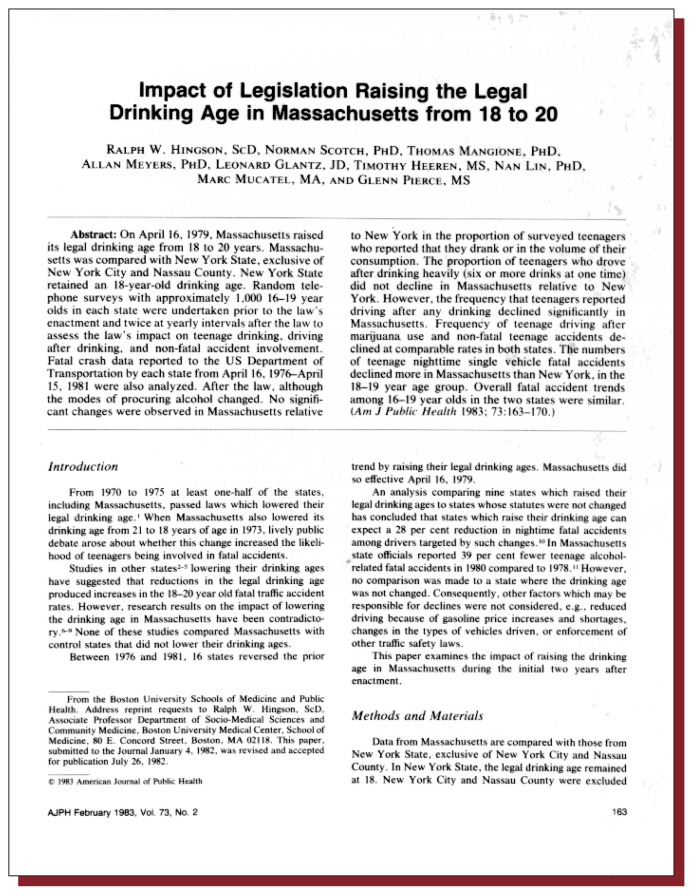
Hingson, R.W.; Scotch, N.; Mangione, T.; Meyers, A.; Glantz, L.; Heeren, T.; Lin, N.; Mucatel, M.; and Pierce, G. Impact of legislation raising the legal drinking age in Massachusetts from 18 to 20. *American Journal of Public Health* 73(2):163–170, 1983.

This study examining the impact of increasing the minimum legal drinking age (MLDA) in Massachusetts beautifully illustrates the complexity of measuring the effects of changes in public policy in the midst of other concurrent changes and historical trends. Hingson and colleagues performed their study during a time of transition, when some States had implemented increases in the drinking age whereas others had not. Moreover, States with an increased MLDA already in effect had not been studied extensively using longitudinal evaluation; thus, many unanswered questions remained. For example: How would MLDA’s eventually affect drinking levels of different age groups? How would the differences in MLDA affect population subgroups such as women? And how would differences in MLDA’s of adjacent States affect regional drinking behavior?

When Hingson and colleagues undertook this study, there was a great need for objective, comprehensive evaluation of how, and if, raising the MLDA would affect drinking behavior and, more specifically, drinking and driving behavior among young people. Since their study, more research has been conducted on this topic. And although many public health and traffic safety experts today accept that the decrease in alcohol-related motor vehicle fatalities for young drivers was causally related to nationwide increases in the MLDA (e.g., Fell 1990; O’Malley and Wagenaar 1991; Jones et al. 1992; Wagenaar 1993; Chaloupka et al. 1993), other experts question whether these increases in MLDA actually had a measurable, beneficial effect on traffic safety (e.g., Asch and Levy 1990; Mooney et al. 1992).

Hingson and colleagues compared the drinking behavior of teenagers in Massachusetts with those in New York (excluding New York City and Nassau County). The two States had similar critical variables, such as laws concerning the age at which drivers could obtain their drivers’ licenses and penalties for driving while intoxicated as well as similar weather conditions. However, Massachusetts raised the MLDA from age 18 to 20 and New York retained age 18 as the MLDA. In the study, researchers compiled data from telephone surveys, crash data, arrest data, and interviews with law enforcement and other officials. They found that after raising the MLDA, the frequency of teen drinking in bars and clubs and the percentage of teens likely to purchase alcohol in liquor and grocery stores declined in Massachusetts, compared with New York. Still, a surprising 40 percent of the Massachusetts teenagers surveyed reported that they had attempted to purchase alcohol after the legislative change. Of the teenagers who continued to purchase alcohol, one-third were never asked for identification, and very few were arrested. Other Massachusetts teenagers readily adapted to the new law by getting older friends to purchase alcohol for them. When asked about drinking and driving, teenagers from Massachusetts and New York reported similar frequencies of driving after heavy drinking (i.e., after consuming six or more drinks). However, the number of teenagers who reported that they drove after any drinking declined significantly more in Massachusetts.

Hingson and colleagues analyzed survey data on drinking, driving after drinking, and nonfatal accidents and found that the incidence of drinking and driving among older Massachusetts teenagers (i.e., 18 and 19 years old), who had previously been entitled to drink, was not significantly different from drinking and driving among younger teenagers (i.e., 16 and 17 years old). Nighttime single vehicle fatal crashes showed a greater decline for Massachusetts teenagers ages 18 and 19 than for teenagers of the same ages in New York, but no differences were found for younger teenagers in both States.

In their article, Hingson and colleagues noted that by raising the MLDA, officials in the State of Massachusetts provided a “symbolic statement” to teenagers that the citizens of that State disapproved of teen drinking and feared that accidents may result as a consequence of teen drinking. However, the researchers also acknowledged that enforcement of the new MLDA law was uneven across Massachusetts, with many police officers and other officials reluctant to impose sanctions. Police interviews conducted by Hingson and colleagues revealed that lack of personnel and competing priorities were the reasons most often cited for the uneven enforcement. Other comments offered by police officers showed that many of them did not heartily support the law. Indeed, the researchers reported that many officers did not perceive teenage purchasing of alcohol or drinking as a “sufficiently serious crime to stigmatize juveniles by putting an arrest on their records.” Enforcement of sanctions against retailers who sold to underage customers was even more lax; liquor license revocations by the State did not increase in frequency after the law’s passage.

Hingson and colleagues’ study focused on only a single State, with a second State used as a comparison. Subsequent studies combining larger numbers of States appear to show clearly the impact of increases in the MLDA in reducing teenage drinking and driving and involvement in alcohol-related crashes. Hingson and colleagues also were among the first to raise cautionary flags concerning interpretation of the impact of such strategies in reducing nonfatal and fatal crashes. As the researchers pointed out, it is not sufficient simply to enact legislation. If the public and, in particular, the law enforcement community, do not completely support the legislation, its effectiveness is diluted.

The study performed by Hingson and colleagues exemplified the limitations of one strategy in reducing drinking and alcohol-related crashes among teenagers. Strategies designed to reduce drinking and driving are affected by and, in turn, affect the public’s attitude toward drivers who drink. The gradual improvement seen in traffic safety is undoubtedly a function of many strategies that have reinforced each other to bring about change.
